# Supplementary material for: An ecosyndemic framework for understanding obesity: Spatial clustering of health, environmental and socioeconomic disadvantage in the Netherlands
Source: PLoS One. 2026 Aug 14;21(8):e0354964. doi: 10.1371/journal.pone.0354964 (PMC13475877; doi:10.1371/journal.pone.0354964)
Supplement: Supplementary file 3 [file pone.0354964.s003.docx]

**Supplementary File 3: Factor loadings on principal components**

| PCA loadings | | | | | |
| --- | --- | --- | --- | --- | --- |
| Variable | PC1 | PC2 | PC3 | PC4 | PC5 |
| SES-WOA | 0.338 | 0.192 | −0.263 | −0.676 | 0.259 |
| Obesity | −0.158 | −0.631 | −0.062 | 0.318 | 0.455 |
| Chronic disease | −0.229 | −0.538 | −0.096 | −0.560 | 0.167 |
| Risk of anxiety and/or depression | −0.342 | −0.256 | 0.169 | −0.279 | −0.728 |
| Bikeability | 0.291 | −0.114 | 0.785 | −0.089 | 0.139 |
| Walkability | 0.424 | −0.200 | 0.264 | −0.064 | −0.052 |
| Drivability | 0.402 | −0.245 | −0.070 | 0.020 | −0.172 |
| Density of sport facilitations | −0.386 | 0.200 | 0.136 | −0.140 | 0.264 |
| Healthiness food environment | 0.341 | −0.239 | −0.425 | 0.135 | −0.216 |
