# Supplementary material for: An ecosyndemic framework for understanding obesity: Spatial clustering of health, environmental and socioeconomic disadvantage in the Netherlands
Source: PLoS One. 2026 Aug 14;21(8):e0354964. doi: 10.1371/journal.pone.0354964 (PMC13475877; doi:10.1371/journal.pone.0354964)
Supplement: Supplementary file 6 [file pone.0354964.s006.docx]

**Supplementary File 6: Sensitivity analysis clusters**

**Contingency table of shift in neighbourhoods to other classifications.**

Towards privileged Anxiety/depression - low obesity Ecosyndemic Syndemic Privileged

Anxiety/depression - low obesity	1	969	12	9	0

Ecosyndemic	0	21	980	69	0

Privileged	232	0	0	82	6111

Syndemic	89	0	95	1618	34

Towards privileged	1872	52	0	170	4
