# Supplementary material for: An ecosyndemic framework for understanding obesity: Spatial clustering of health, environmental and socioeconomic disadvantage in the Netherlands
Source: PLoS One. 2026 Aug 14;21(8):e0354964. doi: 10.1371/journal.pone.0354964 (PMC13475877; doi:10.1371/journal.pone.0354964)
Supplement: Supplementary file 7 [file pone.0354964.s008.docx]

**Response to reviewers**

Reviewer #1: Thank you for the opportunity to review the manuscript entitled “An ecosyndemic framework for understanding obesity: spatial clustering of health, environmental and socioeconomic disadvantage in the Netherlands”. The authors clearly explain why their research is important (obesity and related health consequences are increasing but vary across space) and the gap that they are addressing (lack of an integrative approach that addresses both neighborhood-level socioeconomic and environmental factors). The authors provide a detailed description of their variables and methods and they have conducted a thorough statistical analysis. The map that they have developed can indeed be a practical guide for policy makers and others. Taken together, it can be argued that this is a very relevant study that certainly deserves publication. However, some minor issues remains:
-The authors state that the metrics indicated that two or three clusters would suffice, but that due to ‘model interpretability, policy relevance and diversity of profiles’ five clusters were deployed in the research. This explanation is a bit thin. What is the added value of selecting five clusters over two or three? What is missing if the authors would select four instead of five clusters? Also because psychological vulnerability becomes quite central to the paper, by being a separate cluster but it is not clear why (as the paper’s main focus is on obesity and it’s consequences, so why generate a separate cluster on psychological vulnerability with low obesity scores?).
-The authors could elaborate on their measurement of walkability, bikeability and drivability. Were the metrics confined to the administrative neighborhood boundaries? Or were these factors measured within a certain range from the center of the administrative neighborhood? And what formed the basis for this decision? The authors acknowledge in their discussion section that administrative neighborhood boundaries have some limitations, so it is useful for the reader to know what considerations were taken into account. Also because calculating walkability, bikeability, and drivability around a center point instead of confining it to administrative borders, might be a more accurate resemblance of the daily lives of inhabitants.

Response: We thank the reviewer for the time taken to review the paper and for the thoughtful comments.

Regarding the first issue: We agree with the reviewer that the rationale for selecting five clusters required further clarification. We have revised the manuscript to provide a more detailed explanation of the cluster selection process. Although the clustering diagnostics suggested that two- and three-cluster solutions were the most parsimonious representations of the data, both the elbow method and the gap statistic identified a five-cluster solution as a plausible alternative. In contrast, a four-cluster solution was not supported by either diagnostic. We therefore selected the five-cluster solution because it remained supported by the clustering diagnostics while providing greater differentiation between neighborhood profiles. Following characterization of the resulting clusters, one cluster emerged that was characterized by elevated mental health vulnerability despite relatively favorable obesity outcomes. This profile would likely have been less visible in more aggregated clustering solutions and contributed to the interpretability and policy relevance of the identified neighborhood typologies. The sentence in the first paragraph of the results section has been amended to:

“The elbow method and gap statistic supported two-, three-, and five-cluster solutions (S4 and S5 Figs), whereas a four-cluster solution was not identified as optimal by either diagnostic. We selected the five-cluster solution, as it offered greater differentiation between neighborhood profiles and enhanced policy relevance while remaining supported by clustering diagnostics.

Regarding the second issue: We thank the reviewer for this helpful suggestion. We have expanded the Methods section to clarify how walkability, bikeability and drivability were operationalized. These indicators were derived from the Obesogenic Built Environment CharacterisTics (OBCT) index developed by Lam et al. These indicators were operationalized at the level of Dutch administrative neighborhoods using the 2016 neighborhood boundaries. Although some underlying environmental measures were based on spatial density and proximity calculations, the resulting walkability, bikeability and drivability scores reflect neighborhood-level characteristics rather than exposures around a neighborhood centroid or individual activity spaces. The methods section now reads: The revised paragraph:

“Nine neighborhood-level variables were used as input for the clustering analyses (Table 1). Indicators were selected based on the ecosyndemic framework and existing evidence linking socioeconomic conditions, obesogenic environments and health outcomes. Obesity, chronic disease and risk of anxiety/depression were included because they represent interrelated physical and mental health burdens that frequently co-occur and contribute to population health inequalities, these were derived from the Dutch Health Monitor. SEP was assessed using SES-WOA, a composite score including wealth, educational level and recent employment, and was included as a key contextual determinant of health and a central driver of syndemic and ecosyndemic processes (Statistics Netherlands, 2026). Environmental indicators were derived from the validated Obesogenic Built Environment CharacterisTics (OBCT) index developed by Lam et al., which has previously been used to characterize neighborhood environments relevant for dietary behaviors and physical activity (Lam et al., 2023). These neighborhood-level indicators were derived using the 2016 Dutch administrative neighborhood boundaries and capture characteristics of the local food and physical activity environment, including walkability, bikeability, driveability and the food environment. All construct scores were scaled so that higher values represent more obesogenic neighborhood characteristics, whereas lower values indicate more favourable, health-promoting environments. Together, the clustering variables capture complementary dimensions of the social, environmental and health domains central to the ecosyndemic framework.”

Reviewer #2: The research uses an ecosyndemic approach to characterize obesogenic neighborhoods by looking into their different situations regarding health, environmental features, and socioeconomic position. The results show very interesting combinations of neighborhood characteristics and their spatial distribution. The results also provide clear evidence for policymaking. Only a few revisions will be needed to make the paper even more reader-friendly.

Response: We thank the reviewer for the time taken to review the paper and for the thoughtful comments.

1. Introduction: The authors introduce the concepts of syndemics and ecosyndemics in the introduction. The three bullet points after the mention of syndemics seem to link environmental and social factors to the cluster of health issues in an abstract way. On the basis of the three very important bullet points, the authors can consider elaborating more on what the three bullet points mean, listing some empirical evidence of the three mechanisms/descriptions in them, or giving brief examples/cases of how they work. Some of the detailed explanations of the concepts and mechanisms seem to be in the discussion section. The authors may consider mentioning some of them in the introduction section, so that the readers can understand the context earlier.

Response: We thank the reviewer for this helpful suggestion. We agree that the concepts of syndemics and ecosyndemics could be illustrated more clearly in the introduction. We have therefore added a short explanation and practical example showing how socioeconomic disadvantage, obesogenic environments, obesity and mental health problems may interact and reinforce one another within neighborhoods. This addition aims to make the underlying mechanisms of the ecosyndemic framework more accessible to readers before presenting the study objectives. We added the following to the third paragraph of the introduction:

“For example, residents of socioeconomically disadvantaged neighborhoods may simultaneously experience limited access to healthy foods, fewer opportunities for physical activity, financial stress and poorer mental health. These conditions and exposures may reinforce one another. Chronic stress can contribute to unhealthy dietary behaviours and obesity, while obesity and chronic disease may in turn negatively affect mental wellbeing. Such interactions exemplify how social and environmental conditions can contribute to the clustering and amplification of health problems within specific populations.”

2. Operationalisation: In section 2.2, the authors introduce the indicators of SEP, health, and the obesogenic index. There is a lot of information in the indicators and in Table 1 that is related to the research context and can be elaborated on. For example, when revising the introduction section, the authors may also consider introducing some existing literature about the impact and relevance of these indicators, so that the readers can get prepared in advance and understand more easily why these specific indicators are chosen in this research. In the research study section, the authors can also consider briefly explaining directly why these indicators/data are chosen, such as for theoretical need, for data accessibility, etc., whether they have been used in other studies, and their performance in those studies.

Response: We thank the reviewer for this helpful suggestion. We agree that the rationale for selecting the clustering indicators could be made more explicit. We therefore expanded the “clustering variables” section to clarify that the indicators were selected based on the ecosyndemic framework and existing evidence linking socioeconomic disadvantage, obesogenic environments and health outcomes. We now explicitly explain the inclusion of obesity, chronic disease and anxiety/depression risk as complementary health indicators, the role of SEP as a key contextual determinant of health, and the use of the validated OBCT index developed by Lam et al. to characterize neighborhood obesogenic environments. These additions provide readers with greater context regarding the theoretical and empirical relevance of the selected indicators. The revised paragraph:

“Nine neighborhood-level variables were used as input for the clustering analyses (Table 1). Indicators were selected based on the ecosyndemic framework and existing evidence linking socioeconomic conditions, obesogenic environments and health outcomes. Obesity, chronic disease and risk of anxiety/depression were included because they represent interrelated physical and mental health burdens that frequently co-occur and contribute to population health inequalities, these were derived from the Dutch Health Monitor. SEP was assessed using SES-WOA, a composite score including wealth, educational level and recent employment, and was included as a key contextual determinant of health and a central driver of syndemic and ecosyndemic processes (Statistics Netherlands, 2026). Environmental indicators were derived from the validated Obesogenic Built Environment CharacterisTics (OBCT) index developed by Lam et al., which has previously been used to characterize neighborhood environments relevant for dietary behaviors and physical activity (Lam et al., 2023). These neighborhood-level indicators were derived using the 2016 Dutch administrative neighborhood boundaries and capture characteristics of the local food and physical activity environment, including walkability, bikeability, driveability and the food environment. All construct scores were scaled so that higher values represent more obesogenic neighborhood characteristics, whereas lower values indicate more favourable, health-promoting environments. Together, the clustering variables capture complementary dimensions of the social, environmental and health domains central to the ecosyndemic framework.”

Visualisation: The results and discussion are clear and fruitful. The authors may consider visualising the characteristics of each type of neighborhood in the results, so that the readers can grasp the main point of each type more quickly and easily. For example, for each type of neighborhood, the authors can draw a set of charts to describe the combination of the scores/percentages in their indicators. By putting the five sets of charts together, the readers could understand more directly what the main differences between them are, and what the main problems/features of each of them are.

Response: We thank the reviewer for this helpful suggestion. To facilitate interpretation of the identified neighborhood typologies, we added a new figure visualizing the standardized values of the clustering indicators across the five clusters. This figure provides a concise overview of the distinguishing characteristics of each neighborhood type and complements the detailed numerical results presented in Table 2.

*Fig 1: Cluster profiles across the variables used for the clustering of neighborhoods. Colors represent standardized values oriented such that red indicates a relatively less favorable profiles and blue indicates a relatively more favorable profile. Values shown within cells represent cluster-specific median value. Health indicators include obesity prevalence (%), chronic disease prevalence (%) and moderate/high risk of anxiety and depression (%). Socioeconomic position is represented by the SES-WOA score. Environmental indicators include walkability, bikeability, driveability and sports facility density (expressed as z-scores), and food environmental quality (FEHI score).*

Fig 1: Cluster profiles across the variables used for the clustering of neighborhoods.
