# Supplementary material for: An ecosyndemic framework for understanding obesity: Spatial clustering of health, environmental and socioeconomic disadvantage in the Netherlands
Source: PLoS One. 2026 Aug 14;21(8):e0354964. doi: 10.1371/journal.pone.0354964 (PMC13475877; doi:10.1371/journal.pone.0354964)
Supplement: Supplementary file 9 [file pone.0354964.s004.docx]

**Supplementary File 4: Elbow method to determine optimal number of clusters**
