# Supplementary material for: An ecosyndemic framework for understanding obesity: Spatial clustering of health, environmental and socioeconomic disadvantage in the Netherlands
Source: PLoS One. 2026 Aug 14;21(8):e0354964. doi: 10.1371/journal.pone.0354964 (PMC13475877; doi:10.1371/journal.pone.0354964)
Supplement: Peer review history

# Peer review: Decision Letter 0

**DOI:** 10.1371/journal.pone.0354964.r001

**Authors:** Floris Vermeulen

1 Jul 2026

Dear Dr. Muilwijk,

plosone@plos.org. When you're ready to submit your revision, log on to https://www.editorialmanager.com/pone/ and select the 'Submissions Needing Revision' folder to locate your manuscript file.

- A letter that responds to each point raised by the academic editor and reviewer(s). You should upload this letter as a separate file labeled 'Response to Reviewers'.
- A marked-up copy of your manuscript that highlights changes made to the original version. You should upload this as a separate file labeled 'Revised Manuscript with Track Changes'.
- An unmarked version of your revised paper without tracked changes. You should upload this as a separate file labeled 'Manuscript'.

If you would like to make changes to your financial disclosure, please include your updated statement in your cover letter. Guidelines for resubmitting your figure files are available below the reviewer comments at the end of this letter.

If applicable, we recommend that you deposit your laboratory protocols in protocols.io to enhance the reproducibility of your results. Protocols.io assigns your protocol its own identifier (DOI) so that it can be cited independently in the future. For instructions see: https://journals.plos.org/plosone/s/submission-guidelines#loc-laboratory-protocols. Additionally, PLOS ONE offers an option for publishing peer-reviewed Lab Protocol articles, which describe protocols hosted on protocols.io. Read more information on sharing protocols at https://plos.org/protocols?utm_medium=editorial-email&utm_source=authorletters&utm_campaign=protocols.

As the corresponding author, your ORCID iD is verified in the submission system and will appear in the published article. PLOS supports the use of ORCID, and we encourage all coauthors to register for an ORCID iD and use it as well. Please encourage your coauthors to verify their ORCID iD within the submission system before final acceptance, as unverified ORCID iDs will not appear in the published article. *Only*  the individual author can complete the verification step; PLOS staff *cannot*  verify ORCID iDs on behalf of authors.

We look forward to receiving your revised manuscript.

Kind regards,

Floris Vermeulen

Academic Editor

PLOS One

Journal requirements:

When submitting your revision, we need you to address these additional requirements.

1. Please ensure that your manuscript meets PLOS ONE's style requirements, including those for file naming. The PLOS ONE style templates can be found at

https://journals.plos.org/plosone/s/file?id=wjVg/PLOSOne_formatting_sample_main_body.pdf and

https://journals.plos.org/plosone/s/file?id=ba62/PLOSOne_formatting_sample_title_authors_affiliations.pdf

2. Thank you for stating the following financial disclosure:

“This project received funding from the Netherlands Organization for Health Research and Development (ZonMW), grant no: 05550032110023. This publication is part of the project “ECOTIP” (with project number NWA.1518.22.151) of the research programme “Dutch Research Agenda Routes by Consortia” (NWA-ORC 2022) which is financed by the Dutch Research Council (NWO).”

Please state what role the funders took in the study.  If the funders had no role, please state: "The funders had no role in study design, data collection and analysis, decision to publish, or preparation of the manuscript."

If this statement is not correct you must amend it as needed.

Please include this amended Role of Funder statement in your cover letter; we will change the online submission form on your behalf.

3. Thank you for stating the following in the Funding Section of your manuscript:

“This project received funding from the Netherlands Organization for Health Research and Development (ZonMW), grant no: 05550032110023. This publication is part of the project “ECOTIP” (with project number NWA.1518.22.151) of the research programme “Dutch Research Agenda Routes by Consortia” (NWA-ORC 2022) which is financed by the Dutch Research Council (NWO).”

We note that you have provided funding information that is currently declared in your Funding Statement. However, funding information should not appear in the Acknowledgments section or other areas of your manuscript. We will only publish funding information present in the Funding Statement section of the online submission form.

Please remove any funding-related text from the manuscript and let us know how you would like to update your Funding Statement. Currently, your Funding Statement reads as follows:

“This project received funding from the Netherlands Organization for Health Research and Development (ZonMW), grant no: 05550032110023. This publication is part of the project “ECOTIP” (with project number NWA.1518.22.151) of the research programme “Dutch Research Agenda Routes by Consortia” (NWA-ORC 2022) which is financed by the Dutch Research Council (NWO).”

Please include your amended statements within your cover letter; we will change the online submission form on your behalf.

4. We note that you have indicated that there are restrictions to data sharing for this study. For studies involving human research participant data or other sensitive data, we encourage authors to share de-identified or anonymized data. However, when data cannot be publicly shared for ethical reasons, we allow authors to make their data sets available upon request. For information on unacceptable data access restrictions, please see http://journals.plos.org/plosone/s/data-availability#loc-unacceptable-data-access-restrictions.

Before we proceed with your manuscript, please address the following prompts:

a) If there are ethical or legal restrictions on sharing a de-identified data set, please explain them in detail (e.g., data contain potentially identifying or sensitive patient information, data are owned by a third-party organization, etc.) and who has imposed them (e.g., a Research Ethics Committee or Institutional Review Board, etc.). Please also provide contact information for a data access committee, ethics committee, or other institutional body to which data requests may be sent.

b) If there are no restrictions, please upload the minimal anonymized data set necessary to replicate your study findings to a stable, public repository and provide us with the relevant URLs, DOIs, or accession numbers. Please see http://www.bmj.com/content/340/bmj.c181.long for guidelines on how to de-identify and prepare clinical data for publication. For a list of recommended repositories, please see https://journals.plos.org/plosone/s/recommended-repositories. You also have the option of uploading the data as Supporting Information files, but we would recommend depositing data directly to a data repository if possible.

Please update your Data Availability statement in the submission form accordingly.

5. Please include captions for your Supporting Information files at the end of your manuscript, and update any in-text citations to match accordingly. Please see our Supporting Information guidelines for more information: http://journals.plos.org/plosone/s/supporting-information.

If the reviewer comments include a recommendation to cite specific previously published works, please review and evaluate these publications to determine whether they are relevant and should be cited. There is no requirement to cite these works unless the editor has indicated otherwise.

Please review your reference list to ensure that it is complete and correct. If you have cited papers that have been retracted, please include the rationale for doing so in the manuscript text, or remove these references and replace them with relevant current references. Any changes to the reference list should be mentioned in the rebuttal letter that accompanies your revised manuscript. If you need to cite a retracted article, indicate the article’s retracted status in the References list and also include a citation and full reference for the retraction notice.

[Note: HTML markup is below. Please do not edit.]

Reviewers' comments:

Reviewer's Responses to Questions

**Comments to the Author**

1. Is the manuscript technically sound, and do the data support the conclusions?

Reviewer #1: Yes

Reviewer #2: Yes

**********

2. Has the statistical analysis been performed appropriately and rigorously?

Reviewer #1: Yes

Reviewer #2: Yes

**********

3. Have the authors made all data underlying the findings in their manuscript fully available?

The PLOS Data policy

Reviewer #1: Yes

Reviewer #2: Yes

**********

4. Is the manuscript presented in an intelligible fashion and written in standard English?

Reviewer #1: Yes

Reviewer #2: Yes

**********

Reviewer #1: Thank you for the opportunity to review the manuscript entitled “An ecosyndemic framework for understanding obesity: spatial clustering of health, environmental and socioeconomic disadvantage in the Netherlands”. The authors clearly explain why their research is important (obesity and related health consequences are increasing but vary across space) and the gap that they are addressing (lack of an integrative approach that addresses both neighborhood-level socioeconomic and environmental factors). The authors provide a detailed description of their variables and methods and they have conducted a thorough statistical analysis. The map that they have developed can indeed be a practical guide for policy makers and others. Taken together, it can be argued that this is a very relevant study that certainly deserves publication. However, some minor issues remains:

-The authors state that the metrics indicated that two or three clusters would suffice, but that due to ‘model interpretability, policy relevance and diversity of profiles’ five clusters were deployed in the research. This explanation is a bit thin. What is the added value of selecting five clusters over two or three? What is missing if the authors would select four instead of five clusters? Also because psychological vulnerability becomes quite central to the paper, by being a separate cluster but it is not clear why (as the paper’s main focus is on obesity and it’s consequences, so why generate a separate cluster on psychological vulnerability with low obesity scores?).

-The authors could elaborate on their measurement of walkability, bikeability and drivability. Were the metrics confined to the administrative neighborhood boundaries? Or were these factors measured within a certain range from the center of the administrative neighborhood? And what formed the basis for this decision? The authors acknowledge in their discussion section that administrative neighborhood boundaries have some limitations, so it is useful for the reader to know what considerations were taken into account. Also because calculating walkability, bikeability, and drivability around a center point instead of confining it to administrative borders, might be a more accurate resemblance of the daily lives of inhabitants.

Reviewer #2: The research uses an ecosyndemic approach to characterize obesogenic neighborhoods by looking into their different situations regarding health, environmental features, and socioeconomic position. The results show very interesting combinations of neighbourhood characteristics and their spatial distribution. The results also provide clear evidence for policymaking. Only a few revisions will be needed to make the paper even more reader-friendly.

1. Introduction: The authors introduce the concepts of syndemics and ecosyndemics in the introduction. The three bullet points after the mention of syndemics seem to link environmental and social factors to the cluster of health issues in an abstract way. On the basis of the three very important bullet points, the authors can consider elaborating more on what the three bullet points mean, listing some empirical evidence of the three mechanisms/descriptions in them, or giving brief examples/cases of how they work. Some of the detailed explanations of the concepts and mechanisms seem to be in the discussion section. The authors may consider mentioning some of them in the introduction section, so that the readers can understand the context earlier.

2. Operationalisation: In section 2.2, the authors introduce the indicators of SEP, health, and the obesogenic index. There is a lot of information in the indicators and in Table 1 that is related to the research context and can be elaborated on. For example, when revising the introduction section, the authors may also consider introducing some existing literature about the impact and relevance of these indicators, so that the readers can get prepared in advance and understand more easily why these specific indicators are chosen in this research. In the research study section, the authors can also consider briefly explaining directly why these indicators/data are chosen, such as for theoretical need, for data accessibility, etc., whether they have been used in other studies, and their performance in those studies.

3. Visualisation: The results and discussion are clear and fruitful. The authors may consider visualising the characteristics of each type of neighbourhood in the results, so that the readers can grasp the main point of each type more quickly and easily. For example, for each type of neighbourhood, the authors can draw a set of charts to describe the combination of the scores/percentages in their indicators. By putting the five sets of charts together, the readers could understand more directly what the main differences between them are, and what the main problems/features of each of them are.

**********

what does this mean?). If published, this will include your full peer review and any attached files.

If you choose “no”, your identity will remain anonymous but your review may still be made public.

**Do you want your identity to be public for this peer review?**  For information about this choice, including consent withdrawal, please see our Privacy Policy

Reviewer #1: No

Reviewer #2: No

**********

[NOTE: If reviewer comments were submitted as an attachment file, they will be attached to this email and accessible via the submission site. Please log into your account, locate the manuscript record, and check for the action link "View Attachments". If this link does not appear, there are no attachment files.]

To ensure your figures meet our technical requirements, please review our figure guidelines: https://journals.plos.org/plosone/s/figures

You may also use PLOS’s free figure tool, NAAS, to help you prepare publication quality figures: https://journals.plos.org/plosone/s/figures#loc-tools-for-figure-preparation.

NAAS will assess whether your figures meet our technical requirements by comparing each figure against our figure specifications.

# Author comment: Author response to Decision Letter 1

**DOI:** 10.1371/journal.pone.0354964.r002

14 Jul 2026

Reviewer #1: Thank you for the opportunity to review the manuscript entitled “An ecosyndemic framework for understanding obesity: spatial clustering of health, environmental and socioeconomic disadvantage in the Netherlands”. The authors clearly explain why their research is important (obesity and related health consequences are increasing but vary across space) and the gap that they are addressing (lack of an integrative approach that addresses both neighborhood-level socioeconomic and environmental factors). The authors provide a detailed description of their variables and methods and they have conducted a thorough statistical analysis. The map that they have developed can indeed be a practical guide for policy makers and others. Taken together, it can be argued that this is a very relevant study that certainly deserves publication. However, some minor issues remains:

-The authors state that the metrics indicated that two or three clusters would suffice, but that due to ‘model interpretability, policy relevance and diversity of profiles’ five clusters were deployed in the research. This explanation is a bit thin. What is the added value of selecting five clusters over two or three? What is missing if the authors would select four instead of five clusters? Also because psychological vulnerability becomes quite central to the paper, by being a separate cluster but it is not clear why (as the paper’s main focus is on obesity and it’s consequences, so why generate a separate cluster on psychological vulnerability with low obesity scores?).

-The authors could elaborate on their measurement of walkability, bikeability and drivability. Were the metrics confined to the administrative neighborhood boundaries? Or were these factors measured within a certain range from the center of the administrative neighborhood? And what formed the basis for this decision? The authors acknowledge in their discussion section that administrative neighborhood boundaries have some limitations, so it is useful for the reader to know what considerations were taken into account. Also because calculating walkability, bikeability, and drivability around a center point instead of confining it to administrative borders, might be a more accurate resemblance of the daily lives of inhabitants.

Response: We thank the reviewer for the time taken to review the paper and for the thoughtful comments.

Regarding the first issue: We agree with the reviewer that the rationale for selecting five clusters required further clarification. We have revised the manuscript to provide a more detailed explanation of the cluster selection process. Although the clustering diagnostics suggested that two- and three-cluster solutions were the most parsimonious representations of the data, both the elbow method and the gap statistic identified a five-cluster solution as a plausible alternative. In contrast, a four-cluster solution was not supported by either diagnostic. We therefore selected the five-cluster solution because it remained supported by the clustering diagnostics while providing greater differentiation between neighborhood profiles. Following characterization of the resulting clusters, one cluster emerged that was characterized by elevated mental health vulnerability despite relatively favorable obesity outcomes. This profile would likely have been less visible in more aggregated clustering solutions and contributed to the interpretability and policy relevance of the identified neighborhood typologies. The sentence in the first paragraph of the results section has been amended to:

“The elbow method and gap statistic supported two-, three-, and five-cluster solutions (S4 and S5 Figs), whereas a four-cluster solution was not identified as optimal by either diagnostic. We selected the five-cluster solution, as it offered greater differentiation between neighborhood profiles and enhanced policy relevance while remaining supported by clustering diagnostics.

Regarding the second issue: We thank the reviewer for this helpful suggestion. We have expanded the Methods section to clarify how walkability, bikeability and drivability were operationalized. These indicators were derived from the Obesogenic Built Environment CharacterisTics (OBCT) index developed by Lam et al. These indicators were operationalized at the level of Dutch administrative neighborhoods using the 2016 neighborhood boundaries. Although some underlying environmental measures were based on spatial density and proximity calculations, the resulting walkability, bikeability and drivability scores reflect neighborhood-level characteristics rather than exposures around a neighborhood centroid or individual activity spaces. The methods section now reads: The revised paragraph:

“Nine neighborhood-level variables were used as input for the clustering analyses (Table 1). Indicators were selected based on the ecosyndemic framework and existing evidence linking socioeconomic conditions, obesogenic environments and health outcomes. Obesity, chronic disease and risk of anxiety/depression were included because they represent interrelated physical and mental health burdens that frequently co-occur and contribute to population health inequalities, these were derived from the Dutch Health Monitor. SEP was assessed using SES-WOA, a composite score including wealth, educational level and recent employment, and was included as a key contextual determinant of health and a central driver of syndemic and ecosyndemic processes (Statistics Netherlands, 2026). Environmental indicators were derived from the validated Obesogenic Built Environment CharacterisTics (OBCT) index developed by Lam et al., which has previously been used to characterize neighborhood environments relevant for dietary behaviors and physical activity (Lam et al., 2023). These neighborhood-level indicators were derived using the 2016 Dutch administrative neighborhood boundaries and capture characteristics of the local food and physical activity environment, including walkability, bikeability, driveability and the food environment. All construct scores were scaled so that higher values represent more obesogenic neighborhood characteristics, whereas lower values indicate more favourable, health-promoting environments. Together, the clustering variables capture complementary dimensions of the social, environmental and health domains central to the ecosyndemic framework.”

Reviewer #2: The research uses an ecosyndemic approach to characterize obesogenic neighborhoods by looking into their different situations regarding health, environmental features, and socioeconomic position. The results show very interesting combinations of neighborhood characteristics and their spatial distribution. The results also provide clear evidence for policymaking. Only a few revisions will be needed to make the paper even more reader-friendly.

Response: We thank the reviewer for the time taken to review the paper and for the thoughtful comments.

1. Introduction: The authors introduce the concepts of syndemics and ecosyndemics in the introduction. The three bullet points after the mention of syndemics seem to link environmental and social factors to the cluster of health issues in an abstract way. On the basis of the three very important bullet points, the authors can consider elaborating more on what the three bullet points mean, listing some empirical evidence of the three mechanisms/descriptions in them, or giving brief examples/cases of how they work. Some of the detailed explanations of the concepts and mechanisms seem to be in the discussion section. The authors may consider mentioning some of them in the introduction section, so that the readers can understand the context earlier.

Response: We thank the reviewer for this helpful suggestion. We agree that the concepts of syndemics and ecosyndemics could be illustrated more clearly in the introduction. We have therefore added a short explanation and practical example showing how socioeconomic disadvantage, obesogenic environments, obesity and mental health problems may interact and reinforce one another within neighborhoods. This addition aims to make the underlying mechanisms of the ecosyndemic framework more accessible to readers before presenting the study objectives. We added the following to the third paragraph of the introduction:

“For example, residents of socioeconomically disadvantaged neighborhoods may simultaneously experience limited access to healthy foods, fewer opportunities for physical activity, financial stress and poorer mental health. These conditions and exposures may reinforce one another. Chronic stress can contribute to unhealthy dietary behaviours and obesity, while obesity and chronic disease may in turn negatively affect mental wellbeing. Such interactions exemplify how social and environmental conditions can contribute to the clustering and amplification of health problems within specific populations.”

2. Operationalisation: In section 2.2, the authors introduce the indicators of SEP, health, and the obesogenic index. There is a lot of information in the indicators and in Table 1 that is related to the research context and can be elaborated on. For example, when revising the introduction section, the authors may also consider introducing some existing literature about the impact and relevance of these indicators, so that the readers can get prepared in advance and understand more easily why these specific indicators are chosen in this research. In the research study section, the authors can also consider briefly explaining directly why these indicators/data are chosen, such as for theoretical need, for data accessibility, etc., whether they have been used in other studies, and their performance in those studies.

Response: We thank the reviewer for this helpful suggestion. We agree that the rationale for selecting the clustering indicators could be made more explicit. We therefore expanded the “clustering variables” section to clarify that the indicators were selected based on the ecosyndemic framework and existing evidence linking socioeconomic disadvantage, obesogenic environments and health outcomes. We now explicitly explain the inclusion of obesity, chronic disease and anxiety/depression risk as complementary health indicators, the role of SEP as a key contextual determinant of health, and the use of the validated OBCT index developed by Lam et al. to characterize neighborhood obesogenic environments. These additions provide readers with greater context regarding the theoretical and empirical relevance of the selected indicators. The revised paragraph:

“Nine neighborhood-level variables were used as input for the clustering analyses (Table 1). Indicators were selected based on the ecosyndemic framework and existing evidence linking socioeconomic conditions, obesogenic environments and health outcomes. Obesity, chronic disease and risk of anxiety/depression were included because they represent interrelated physical and mental health burdens that frequently co-occur and contribute to population health inequalities, these were derived from the Dutch Health Monitor. SEP was assessed using SES-WOA, a composite score including wealth, educational level and recent employment, and was included as a key contextual determinant of health and a central driver of syndemic and ecosyndemic processes (Statistics Netherlands, 2026). Environmental indicators were derived from the validated Obesogenic Built Environment CharacterisTics (OBCT) index developed by Lam et al., which has previously been used to characterize neighborhood environments relevant for dietary behaviors and physical activity (Lam et al., 2023). These neighborhood-level indicators were derived using the 2016 Dutch administrative neighborhood boundaries and capture characteristics of the local food and physical activity environment, including walkability, bikeability, driveability and the food environment. All construct scores were scaled so that higher values represent more obesogenic neighborhood characteristics, whereas lower values indicate more favourable, health-promoting environments. Together, the clustering variables capture complementary dimensions of the social, environmental and health domains central to the ecosyndemic framework.”

Visualisation: The results and discussion are clear and fruitful. The authors may consider visualising the characteristics of each type of neighborhood in the results, so that the readers can grasp the main point of each type more quickly and easily. For example, for each type of neighborhood, the authors can draw a set of charts to describe the combination of the scores/percentages in their indicators. By putting the five sets of charts together, the readers could understand more directly what the main differences between them are, and what the main problems/features of each of them are.

Response: We thank the reviewer for this helpful suggestion. To facilitate interpretation of the identified neighborhood typologies, we added a new figure visualizing the standardized values of the clustering indicators across the five clusters. This figure provides a concise overview of the distinguishing characteristics of each neighborhood type and complements the detailed numerical results presented in Table 2.

Fig 1: Cluster profiles across the variables used for the clustering of neighborhoods. Colors represent standardized values oriented such that red indicates a relatively less favorable profiles and blue indicates a relatively more favorable profile. Values shown within cells represent cluster-specific median value. Health indicators include obesity prevalence (%), chronic disease prevalence (%) and moderate/high risk of anxiety and depression (%). Socioeconomic position is represented by the SES-WOA score. Environmental indicators include walkability, bikeability, driveability and sports facility density (expressed as z-scores), and food environmental quality (FEHI score).

Fig 1: Cluster profiles across the variables used for the clustering of neighborhoods.

Attachment Submitted filename: Response to reviewers.docx

# Editor report: Decision Letter 1

**DOI:** 10.1371/journal.pone.0354964.r003

**Authors:** Floris Vermeulen

15 Jul 2026

An ecosyndemic framework for understanding obesity: spatial clustering of health, environmental and socioeconomic disadvantage in the Netherlands

PONE-D-26-12617R1

Dear Dr. Muilwijk,

We’re pleased to inform you that your manuscript has been judged scientifically suitable for publication and will be formally accepted for publication once it meets all outstanding technical requirements.

Within one week, you’ll receive an e-mail detailing the required amendments. When these have been addressed, you’ll receive a formal acceptance letter and your manuscript will be scheduled for publication.

An invoice will be generated when your article is formally accepted. Please note, if your institution has a publishing partnership with PLOS and your article meets the relevant criteria, all or part of your publication costs will be covered. Please make sure your user information is up-to-date by logging into Editorial Manager at Editorial Manager® and clicking the ‘Update My Information' link at the top of the page. For questions related to billing, please contact billing support.

If your institution or institutions have a press office, please notify them about your upcoming paper to help maximize its impact. If they’ll be preparing press materials, please inform our press team as soon as possible -- no later than 48 hours after receiving the formal acceptance. Your manuscript will remain under strict press embargo until 2 pm Eastern Time on the date of publication. For more information, please contact onepress@plos.org.

Kind regards,

Floris Vermeulen

Academic Editor

PLOS One

Additional Editor Comments (optional):

Reviewers' comments:

# Editor report: Acceptance letter

**DOI:** 10.1371/journal.pone.0354964.r004

**Authors:** Floris Vermeulen

PONE-D-26-12617R1

PLOS One

Dear Dr. Muilwijk,

I'm pleased to inform you that your manuscript has been deemed suitable for publication in PLOS One. Congratulations! Your manuscript is now being handed over to our production team.

At this stage, our production department will prepare your paper for publication. This includes ensuring the following:

* All references, tables, and figures are properly cited

* All relevant supporting information is included in the manuscript submission,

* There are no issues that prevent the paper from being properly typeset

You will receive further instructions from the production team, including instructions on how to review your proof when it is ready. Please keep in mind that we are working through a large volume of accepted articles, so please give us a few days to review your paper and let you know the next and final steps.

Lastly, if your institution or institutions have a press office, please let them know about your upcoming paper now to help maximize its impact. If they'll be preparing press materials, please inform our press team within the next 48 hours. Your manuscript will remain under strict press embargo until 2 pm Eastern Time on the date of publication. For more information, please contact onepress@plos.org.

You will receive an invoice from PLOS for your publication fee after your manuscript has reached the completed accept phase. If you receive an email requesting payment before acceptance or for any other service, this may be a phishing scheme. Learn how to identify phishing emails and protect your accounts at https://explore.plos.org/phishing.

If we can help with anything else, please email us at customercare@plos.org.

Thank you for submitting your work to PLOS ONE and supporting open access.

Kind regards,

PLOS ONE Editorial Office Staff

on behalf of

Dr. Floris Vermeulen

Academic Editor

PLOS One
